# Rational Design of Antigen Incorporation Into Subunit Vaccine Biomaterials Can Enhance Antigen-Specific Immune Responses

**DOI:** 10.3389/fimmu.2020.01547

**Published:** 2020-07-21

**Authors:** Alexandra N. Tsoras, Kong M. Wong, Anant K. Paravastu, Julie A. Champion

**Affiliations:** School of Chemical & Biomolecular Engineering, Atlanta, GA, United States

**Keywords:** peptides, nanoparticles, crosslinking, biomaterials, subunit vaccines, immune cell processing, T cell activation

## Abstract

Peptide subunit vaccines increase safety by reducing the risk of off-target responses and improving the specificity of the induced adaptive immune response. The immunogenicity of most soluble peptides, however, is often insufficient to produce robust and lasting immunity. Many biomaterials and delivery vehicles have been developed for peptide antigens to improve immune response while maintaining specificity. Peptide nanoclusters (PNC) are a subunit peptide vaccine material that has shown potential to increase immunogenicity of peptide antigens. PNC are comprised only of crosslinked peptide antigen and have been synthesized from several peptide antigens as small as 8 amino acids in length. However, as with many peptide vaccine biomaterials, synthesis requires adding residues to the peptide and/or engaging amino acids within the antigen epitope covalently to form a stable material. The impact of antigen modifications made to enable biomaterial incorporation or formation is rarely investigated, since the goal of most studies is to compare the soluble antigen with biomaterial form of antigen. This study investigates PNC as a platform vaccine biomaterial to evaluate how peptide modification and biomaterial formation with different crosslinking chemistries affect epitope-specific immune cell presentation and activation. Several types of PNC were synthesized by desolvation from the model peptide epitope SIINFEKL, which is derived from the immunogenic protein ovalbumin. SIINFEKL was altered to include extra residues on each end, strategically chosen to enable multiple conjugation chemistry options for incorporation into PNC. Several crosslinking methods were used to control which functional groups were used to stabilize the PNC, as well as the reducibility of the crosslinking. These variations were evaluated for immune responses and biodistribution following *in vivo* immunization. All modified antigen formulations still induced comparable immune responses when incorporated into PNC compared to unmodified soluble antigen alone. However, some crosslinking methods led to a significant increase in desirable immune responses while others did not, suggesting that not all PNC were processed the same. These results help guide future peptide vaccine biomaterial design, including PNC and a wide variety of conjugated and self-assembled peptide antigen materials, to maximize and tune the desired immune response.

## Introduction

There are many challenges associated with currently available vaccines, including safety concerns, lack of specificity, and absence of protection against heterogenous pathogenic strains (1, 2). Subunit vaccines are a promising solution that address many of these issues. However, they tend to lack comparable immunogenicity to whole pathogen vaccines and often require multiple boosts and adjuvants (3, 4). Peptides are among the smallest antigenic subunits that can be used as vaccines. Specific sequences in an antigenic protein capable of binding immune cell receptors, known as peptide epitopes, can be identified using a variety of advanced analytical techniques (5–10). These peptides can be administered as vaccines to induce proliferation and differentiation of antigen-specific immune cells for future protection against pathogens containing this antigenic peptide sequence (10).

To increase the immunogenic response to small proteins and peptides, they are often conjugated to or incorporated into other proteins and/or biomaterials (11). Although there are some biomaterials that can encapsulate or adsorb unmodified proteins and peptides (12–14), they can also induce immune responses to the material itself (15–19), or induce tolerance to antigens if a delivery material is used multiple times (20). Therefore, it is beneficial for vaccine formulations to minimize delivery of material that is not the target antigen. Vaccine biomaterials in development must balance the minimization of non-target antigen delivery with antigen modifications necessary to incorporate or form into a biomaterial. Many materials address this challenge by utilizing engineering design to induce structurally ordered, hydrophobically assembled, or electrostatically assembled materials made of mostly a target antigen or an altered variant of it (21–27). Covalent or sequence modification of the antigen is often required to enable its stable incorporation into a material. For large protein antigens, modification may have little effect on specific antigenic epitopes. However, for smaller peptides, modification is more likely to affect the characteristics of the peptide, both physicochemical and antigenic.

Non-biodegradable conjugation or sequence modifications are a potential concern regarding the processing and presentation of the peptides by antigen presenting cells (APCs). Peptide antigens are usually the minimum length that APCs can present to other immune cells on surface-presenting proteins, major histocompatibility complex I, or II (MHC I or MHC II) (28, 29). MHC I proteins present intracellular antigens, and are more restrictive in what length of peptides they are able to present. MHC II proteins present extracellular antigens, and, while less restrictive, still have limits in the length of peptide that can be presented (30). Similarly, peptide length is likely to affect the affinity and specificity of attachment to a T or B cell receptor and, ultimately, activation and proliferation of those cells (31, 32). If modified peptide antigens are cleaved to remove part of the epitope sequence while the modifications remain, or residues are altered to the point that the peptide cannot attach to MHC molecules, the peptides will not be able to activate antigen-specific immune cells or induce protection (31, 33). Similarly, if covalent conjugation chemistry disables the ability of APCs to break down peptides into presentable minimal epitopes, the peptides will not be presented.

Peptide nanoclusters (PNC) are vaccine biomaterials designed to completely eliminate carrier materials or self-assembly sequences and, therefore, avoid off target immune responses. PNC are formed by desolvation of peptide antigens and crosslinking into stabilized clusters in suspension (27). This process can be tuned for many different peptides with different characteristics by choosing optimal desolvation conditions for each peptide to yield nanoclusters in a desired size range. Protein nanoclusters are synthesized the same way, but with larger proteins, and have demonstrated the ability to increase the potency and breadth of immune responses (34, 35). PNC allow for comparatively more specific target antigen delivery. However, their small size and limited amino acid diversity lead to the aforementioned antigen incorporation challenges. A key factor that affects PNC formation and stability is the availability of residues with reactive groups that can be used for crosslinking. While residues in the minimal epitope sequence could be used, crosslinking these residues could compromise peptide processing and presentation by APCs or recognition by T or B cell receptors. Furthermore, every peptide epitope has a different sequence, and each new antigen may require different crosslinking mechanisms depending on the available amino acids. Some epitopes may have too few or no reactive groups that could be used for crosslinking. It would be beneficial to be able to apply a standard modification to each antigen that eliminates the dependence on the antigen sequence for reactive groups to crosslink. Such a modification would provide available reactive groups outside of the minimal epitope, ideally orthogonal from those inside the minimal epitope, that can be used to crosslink and stabilize PNC. There are also different types of crosslinking mechanisms that may affect cellular breakdown of particles, which could affect processing and presentation.

To our knowledge, a systematic evaluation of how peptide modifications and crosslinking chemistry for biomaterial synthesis affect immune cell responses to the desired antigen has not been performed. To address this gap, a model epitope, SIINFEKL, derived from the model protein antigen, ovalbumin, was modified for incorporation into PNC. PNC were synthesized by desolvation using several crosslinking methods for different modes of biomaterial incorporation. With multiple formulations of PNC containing the SIINFEKL epitope, we evaluated how the peptide modifications and different PNC crosslinking schemes affected the strength and type of immune response to SIINFEKL. Differences in dendritic cell maturation and antigen presentation, T cell activation, and biodistribution of PNC were observed.

## Materials and Methods

### Materials and Animals

Peptides (SFK: SIINFEKL, SLS: GKCSIINFEKLCKG) were purchased from Genscript at >95% purity. Tetramethylrhodamine (TAMRA)-labeled versions of the above peptides were purchased from Biomatik at >99% purity with TAMRA conjugated to an additional C-terminal lysine. Trimethylolpropane tris(3-mercaptopropionate) (tri-thiol) crosslinker was purchased from Sigma. Tris(2-maleimidoethyl)amine (tri-maleimide) and tris-(succinimidyl)aminotriacetate (tri-NHS) were purchased from ThermoFisher.

Six to eight week old C57BL/6 mice were purchased from Jackson Laboratories and kept in the Physiological Research Laboratory at Georgia Institute of Technology. Mice were fed a standard diet during all studies except for biodistribution studies, in which they were given an alfalfa-free diet to reduce background fluorescence during imaging. All procedures and care were carried out according to regulations and guidelines approved by the Georgia Institute of Technology Institutional Animal Care and Use Committee (Protocol A100259).

### Nanocluster Synthesis

All PNC were synthesized using desolvation with conditions tuned for the characteristics of each peptide and crosslinker combination. The general process remained the same for all variations, and conditions specific to each PNC variation are described in the (Supplementary Table 1). All peptides were solubilized in hexafluoroisopropanol (HFIP) at 2.5 mg/ml, and 100 μl was added to a 6 ml glass vial. Safety note: HFIP is a hazardous chemical with acute oral, dermal, and vapor inhalation toxicity. All handling was conducted with proper personal protective equipment. Under constant stirring at 400 rpm with a 1 cm stir bar, the desired crosslinker was added at the indicated amount. A specific volume of diethyl ether (DEE) was then added at a rate of 1 ml/min with a syringe pump. The solution reacted under constant mixing for the indicated amount of time for crosslinking stabilization to occur. The solution was then transferred to a centrifuge tube and centrifuged at 18,000 g for 7 min. The supernatant was removed, and the pellet resuspended in water at 1 mg/ml. To ensure full resuspension, the solutions were sonicated with a probe 3–4 times for 1 s on/1 s off at 60% strength. For fluorescent versions of each particle type, 10% TAMRA-labeled SLS was added to the initial peptide solution.

### Nanoparticle Characterization

The size and polydispersity (PDI) of PNC were determined by dynamic light scattering (DLS) (Malvern Zetasizer Nano ZS). To ensure stability during storage in Milli-Q® (MQ) water at 4°C, multiple measurements were taken over several days starting at Day 0 immediately after synthesis. Measurement settings are listed in (Supplementary Table 2). At least 10 batches for each PNC type were synthesized and size and PDI measured to ensure reproducibility.

Yield of PNC synthesis batches were measured with quantitative 1D ^1^H nuclear magnetic resonance (NMR) spectra of resuspended PNC. Particles from each synthesis batch were centrifuged as previously described, and the pellet was allowed to dry overnight in a fume-hood to ensure complete removal of HFIP and DEE. Dried PNC were resuspended in a 200 μl solution of deuterated DMSO (Cambridge Isotope Libraries, Inc.) and 10 mM maleic acid (Alfa Aesar). Maleic acid served as an internal standard for ^1^H NMR intensity. All ^1^H spectra were collected on an 18.8 T Bruker Avance III HD NMR with a 3 mm HCN CryoProbe. The relaxation delay (d1) was set to 20 s to ensure complete spin relaxation, and the pulse width was programmed for 30° pulses (36, 37). Peaks in ^1^H NMR spectra were fit using custom code in Wolfram Mathematica and compared to standard solutions to determine the amount of peptide within PNC batches.

### *In vivo* Immunization and Immunological Assays

For immune response study, 6–8 week old C57/BL16 mice (*N* = 6, 50% female, 50% male) were injected intradermally in each forearm with 30 μl (60 μl total) of 1 mM soluble SIINFEKL, soluble SIINFEKL + 10 mM Poly(I:C) low molecular weight (LMW) adjuvant (Invitrogen), SLS-T PNC, SLS-M PNC, or SLS-N PNC. Three additional mice (2 female, 1 male) were injected with saline as a control. Mice in each group were then boosted with a half dose (15 μl in each forearm) on Day 7 and Day 14. On Day 16, mice were sacrificed and axillary and brachial lymph nodes on both sides and spleens were harvested.

Lymphocytes and splenocytes were obtained by gently breaking up tissues in PBS and straining through a 70 μm cell strainer. Pooled lymph node or spleen cells were washed with 15–20 ml PBS and centrifuged at 4°C, 350 g for 5 min. Spleen cells were resuspended in 1 ml 1X RBC lysis buffer (150 mM ammonium chloride, 10 mM sodium bicarbonate, 1.27 mM EDTA) and incubated for 5–10 min on ice. Lysis was then quenched with 10 ml PBS and spleen cells were centrifuged again at 4°C, 350 g for 5 min. All cells were suspended in PBS and divided for surface marker staining or re-stimulation. Cells used for re-stimulation were centrifuged in a round-bottom 96 well-plate at 4°C, 350 g for 5 min and resuspended in 100 μl culture media (RPMI 1640 + L-glutamine + 25 mM 4-(2-hydroxyethyl)-1-piperazineethanesulfonic acid (HEPES) supplemented with 10% heat inactivated fetal bovine serum and 1% penicillin/streptomycin) with 10^∧^6 cells/well. Media was also supplemented with 1 mg/ml SIINFEKL for 6 h for re-stimulation. For the last 3 h of culture, 1X brefeldin A (Biolegend) was added to the wells. After re-stimulation, cells were stained according to the procedures below.

Cells were stained for DC surface markers, T cell surface markers, or intracellular cytokines according to the following protocol. Cells in a round-bottom 96 well-plate, either in PBS from original organ harvest or culture medium from re-stimulation culture, were centrifuged at 4°C, 350 g for 5 min and resuspended in 100 μl PBS premixed with 5 μl/ml Trustain FcX blocking solution (Biolegend). Cells were incubated for 10 min on ice. Cells were centrifuged again and stained with Zombie Violet or Zombie Aqua Fixable Viability Kit (Biolegend) for 30 min according to manufacturer protocols. Cells were centrifuged and washed with 100 μl sterile 1% bovine serum albumin (BSA) in PBS. Cells were centrifuged and suspended in staining solution for 30 min on ice. Cell staining solution was made for each staining panel by adding all antibody stains to 1% BSA in PBS and then adding directly to wells at 100 μl/well. DCs were stained for CD11c (APC/Cy7, 2.5 μl/well), H-2Kb-SIINFEKL (APC, 1 μl/well), CD86 (PE, 2.5 μl/well) (Biolegend), and MHC II (FITC, 0.5 μl/well) (eBioscience). T cells were stained for CD3 (PerCP, 1 μl/well), CD8 (FITC, 0.313 μl/well), CD4 (APC/Cy7, 0.156 μl/well) (Biolegend), and CD69 (APC, 2 μl/well) (Southern Biotech). After staining, cells were washed with 1% BSA in PBS, centrifuged, and fixed with 100 μl 3.7% formaldehyde in PBS for 45 min on ice. Surface-stained T cells and DCs were centrifuged and resuspended in 200 μl 1% BSA in PBS and stored at 4°C until flow cytometry analysis. If intracellular cytokine staining was performed, cells were centrifuged after fixation in the plate and resuspended in 100 μl permeabilization buffer (eBioscience) with intracellular cytokine staining antibodies anti-IFN-γ (PE, 1.5 μl/well) and anti-TNF-α (PE/Cy7, 1.5 μl/well) (Biolegend), and incubated for 45 min on ice. These cells were then centrifuged in the plate, washed with 100 μl 1% BSA in PBS, and resuspended in 200 μl 1% BSA in PBS for storage at 4°C until flow cytometry analysis. Flow cytometry was performed with a BD LSR Fortessa, with up to 3,000,000 lymphocyte events collected (ensuring all data was collected from 150 μl volume run). Data was analyzed with Flow Jo using the gating strategies shown in Supplementary Figures 1–3.

### Biodistribution

Six to eight week old mice were injected intradermally in each forearm with 30 μl (60 μl total) of 1 mM soluble SIINFEKL (10% TAMRA-labeled) or SLS-T (10% TAMRA-labeled). Mice were split into three end point groups: 4, 24, or 72 h (*N* = 4, 50% female, 50% male). For each group, mice were fluorescently imaged (IVIS Spectrum CT) under anesthesia, and then sacrificed at the indicated time point. Two additional mice were injected with 30 μl saline in each forearm (60 μl total) and sacrificed at 4 h to serve as controls. After sacrifice, axillary and brachial lymph nodes and spleens were harvested, imaged in IVIS Spectrum CT, and then placed into vials with 1.4 mm acid washed zirconium grinding beads (VWR). Lymph nodes were pooled into the same vial with 200 μl PBS, and spleens were placed in vial with 500 μl PBS. Organs were homogenized for 1 min in a FastPrep-24 Automated Homogenizer (MP Biomedicals) 0.150 μl of homogenate in PBS from each mouse's pooled lymph nodes or spleens were added to a 96-well-plate and analyzed for fluorescence using 557/583 nm excitation and emission reading on a plate reader (BioTek Synergy H4 Microplate Reader).

### Statistical Methods

All statistical comparisons made between groups in this manuscript were performed using an unmatched ordinary one-way ANOVA comparison. Tukey's *post-hoc* multiple comparison analysis was performed to compare differences between each group. This analysis was performed using Graphpad Prism software (V8.4). Each group comparison with a *p* < 0.05 were considered significantly different from one another. Statistical differences exist between groups labeled with different letters. Groups that share the same letter are not statistically different from each other.

## Results

### SIINFEKL Modification and Nanocluster Synthesis

Protein and peptide nanocluster synthesis via desolvation and crosslinking has been demonstrated for a number of different antigens (34, 38–40). In this study, a strategic peptide modification was applied to the model epitope SIINFEKL, a well-studied MHC I epitope derived from the protein antigen ovalbumin. The new peptide, named strategically lengthened SIINFEKL (SLS), contains the SIINFEKL sequence with the addition of Gly-Lys-Cys to the N-terminus and Cys-Lys-Gly to the C-terminus (GKCSIINFEKLCKG). This design keeps the minimal epitope in the middle of the sequence, releasable by proteolytic cleavage at the cysteines, while enabling PNC crosslinking via multiple cysteines or lysines using thiol-reactive or amine-reactive crosslinkers. Lysines and cysteines were chosen as flanking residues to enable several forms of crosslinking for stabilization without dependence on minimal epitope residues. In this study, SIINFKEL represented a case where one of the flanking modifications (cysteine) had orthogonal reactivity to the epitope, and one (lysine) did not. The glycines were added as non-reactive, water-soluble residues to increase the length and decrease the likelihood that SLS could be loaded onto MHC I without proteolytic cleavage. Peptides larger than 10 amino acids do not bind to the MHC I loading pocket well (30, 32). To evaluate potential differences in how PNC are processed into minimum peptide epitopes, three different crosslinking mechanisms were used to stabilize PNC after desolvation. Trimethylolpropane tris-3(mercaptopropionate) (tri-thiol) is a homo-trifunctional crosslinker that reacts with thiols to create disulfide bonds. Tris(2-maleimidoethyl)amine (tri-maleimide) also reacts with thiols, creating thioether bonds. Tri-thiol is reducibly reversible and tri-maleimide is not (41, 42). However, both crosslinkers react with cysteines in SLS outside of the minimum epitope, ensuring that any crosslinked residues would be removed after proteolytic cleavage into the minimal SIINFEKL epitope. Tris-(succinimidyl)aminotriacetate (tri-NHS) is a tri-functional amine-reactive crosslinker, and forms non-reducible amide bonds with lysines and the terminal amine in SLS. Tri-NHS can also react with the lysine within SIINFEKL, which could hinder the ability of the peptide to be presented or to bind T cell receptors. However, lysine has been shown not to be an important anchor residue in binding MHC I for effective SIINFEKL presentation (30, 33) and since Tri-NHS forms an amide bond, it is possible for it to be cleaved proteolytically (43). Trifunctional crosslinkers were chosen instead of bifunctional to maximize the amount of crosslinking and increase the likelihood of a fully entangled and stabilized nanocluster.

**Figure 1:**
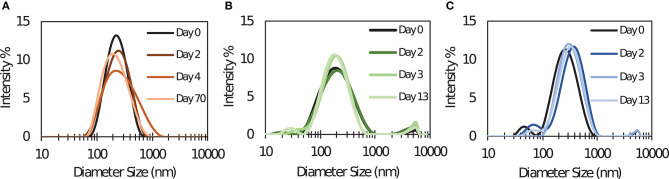
Dynamic light scattering size distribution measurements of **(A)** SLS-T, **(B)** SLS-M, and **(C)** SLS-N PNC taken at several times after synthesis. Distributions over time shown are from one batch, representative of five repeated experiments with different batches of PNC.

SLS PNC were synthesized by desolvation using tri-thiol (SLS-T), tri-maleimide (SLS-M), and tri-NHS (SLS-N) crosslinkers. Although each of these PNC were formed from the same modified SIINFEKL peptide antigen, utilizing different crosslinkers required slight alteration of desolvation conditions to achieve comparable size, PDI, and stability. Table 1 reports particle size and polydispersity index (PDI), which ranged from 184 to 233 nm and 0.189–0.232, respectively (*n* = 10 per PNC type). PNC stability in water at 4°C was evaluated by measuring size over time. Size and PDI remained consistent over several days to weeks as demonstrated by DLS size distributions in Figure 1, so it was expected that changes in size or structure would mainly be due to changing conditions *in vivo*, such as interactions with extracellular proteins or intracellular processing. Size was confirmed and roughly spherical morphology of PNC was observed by Transmission Electron Microscopy (TEM) (Supplementary Figure 4).

**Table 1:** Size, polydispersity, and yield of different SLS PNC formulations.

**PNC**	**Diameter size (nm)**	**Polydispersity index (PDI)**	**Yield (%)**
SLS-T	233 ± 14	0.232 ± 0.035	78.9 ± 9.9
SLS-M	184 ± 15	0.189 ± 0.040	79.3 ± 3.2
SLS-N	205 ± 20	0.225 ± 0.030	78.8 ± 8.3

*n = 10 for size and polydispersity measurements, n = 3 for yield measurements*.

Based on the design of each PNC, SLS-N, and SLS-M particles would likely need to be broken up inside cells proteolytically, whereas the reducing environments inside endo/lysosomes may break up SLS-T PNC. Upon incubating PNC with reducing agents β-mercaptoethanol and DTT, however, it was observed that SLS-T PNC only began to show instability after 24 h incubation with DTT at 37°C (Supplementary Figure 5). Incubation with β-mercaptoethanol for the same time period and temperature did not induce particle instability nor did room temperature incubation with DTT. These results imply that SLS-T PNC are highly crosslinked with disulfide bonds that may have limited accessibility that only allows gradual reducibility. Additionally, SLS-M PNC also showed signs of slight instability with 37°C incubation with DTT. Although thioether bonds are considered non-reducible, maleimide reactions have shown reversibility in some cases (44, 45). SLS-N PNC were unaffected by reducing agents in any conditions and remained stable in size and PDI. This result confirmed that SLS-N PNC were the most likely to require proteolytic cleavage to enable breakup into the minimal epitope.

The yield of SLS peptide incorporated into PNC during the desolvation process was determined to be ~79% for SLS-T, SLS-M, and SLS-N PNC synthesis from measuring three different batches of each PNC (Table 1). These results highlight the consistency of PNC produced by desolvation independent of crosslinking chemistry. Yields were determined by NMR peak integration of ^1^H NMR spectra of PNC resuspended in deuterated DMSO. Peaks around 7.2 ppm were uniquely assigned to the aromatic protons of the phenylalanine sidechain in the SLS peptide (46). By comparing the peak areas in resuspended PNC solutions to a standard solution of unassembled SLS peptide, the total mass of peptide was calculated. The synthesis yield was determined based on comparison to the amount of soluble peptide in the solvent before desolvation. Peak shapes of aromatic protons of the phenylalanine sidechain in all three types of SLS PNC matched those of the unassembled SLS peptide (Supplementary Figure 6). This alignment suggests the crosslinked peptide was well-solvated in DMSO, preventing spin relaxation effects. We also note that large macromolecular assemblies typically exhibit increased peak linewidths due to slower tumbling, complicating quantitative analysis of chemical shift peak areas (47). However, swelling of SLS PNC in DMSO, as indicated by the increase in particle size measured by DLS (Supplementary Figure 7), likely enhanced the mobility of the amino acid sidechains. This solvation of the peptide particles is consistent with a prior molecular dynamics study of α-helical transmembrane peptides where DMSO solvated both hydrophilic and hydrophobic residue sidechains (48). Similar high-resolution quantitative NMR measurements are commonly performed on swollen polymer systems and microplastic particles (49–52). Standard methods of peptide quantification including absorbance and mass spectrometry required large amounts of processing including separation and solvent exchange, which proved challenging and increased measurement error. ^1^H NMR spectroscopy quantified peptide incorporation yields accurately and quickly without the need for separation or extensive processing steps.

### *In vivo* Immune Responses

To assess how peptide antigen modification and biomaterial formulation affected immune responses, SLS-T, SLS-M, and SLS-N PNC formulations were injected intradermally into mice and compared to soluble SIINFEKL antigen and SIINFEKL + poly(I:C) adjuvant injections and a saline control injection. Animals were administered 60 nmol of antigen formulation split into two 30 μl injections; one in each forearm. Intradermal vaccination was chosen because of the significant level of tissue-resident DCs in the skin. This makes the skin a potent target for vaccine delivery compared to muscular or subcutaneous tissue, which do not contain as many DCs (53). Animals were given two half-dose boost injections over 2 weeks and sacrificed on day 16 to harvest draining axillary and brachial lymph nodes and spleens for DC and T cell analysis. Prior to *in vivo* work, cytotoxicity of PNC and SLS peptide in both DC and T cell hybridoma cell lines was evaluated to assess potential toxic side effects *in vivo* (Supplementary Figure 8). Results indicated toxic side effects were not likely based on minimal to no observed cytotoxicity in either cell line.

#### Dendritic Cell Processing

**Figure 2:**
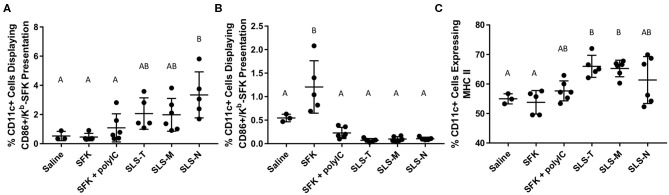
Percent of CD11c+ cells in **(A)** draining lymph nodes and **(B)** spleen co-expressing CD86 and K^b^-SFK. **(C)** Percent of CD11c+ cells in draining lymph nodes expressing MHC II. *N* = 5–6. Differences between letters show significant differences between samples (*p* < 0.05). Samples that share the same letter are not statistically different from each other.

Cellular responses induced by immunization with different antigen formulations varied in several ways. To assess APC processing, CD11c+ DCs in the lymph nodes and spleen were evaluated for MHC I presentation of SIINFEKL and coinciding maturation markers. Figure 2A illustrates that only SLS-N PNC induced a significant increase in co-expression of MHC I presenting SIINFEKL and CD86 in lymph node DCs. Nanoparticles have been shown to have “self-adjuvanting” properties due to their nanoparticulate nature (54–56). This benefit of PNC may explain the increased maturation specifically of SIINFEKL-presenting DCs in mice administered PNC compared to soluble groups, which displayed low levels of maturation. Supplementary Figure 9A shows that of the DCs presenting SIINFEKL, only those in PNC groups have significant surface expression of CD86. It was expected that particles of similar size would have similar DC internalization mechanisms (39, 57). While SLS-T and SLS-M should have similar self-adjuvancy to SLS-N, less efficient, or effective processing of the PNC may have occurred, as they did not induce significantly more co-expression of MHC I presenting SIINFEKL and CD86 than soluble controls (Figure 2A). This suggests that the SLS-N formulation was able to be broken down and processed sufficiently to induce significant levels of DC presentation and maturation. Proteases in endolysosomes and the proteasome in cytosol upon endosomal escape, cleave proteins into minimal epitopes using several mechanisms of amide bond breakage, so it is possible that the amide bonds that form SLS-N PNC are broken down more efficiently by these mechanisms (58, 59) SLS-M is stabilized with thioether bonds, which are commonly used to increase metabolic stability (60–62). Although SLS-T PNC are crosslinked with reversible disulfide bonds, these bonds were shown to be difficult to fully reduce (Supplementary Figure 5) and may lead to decreased ability of DCs to break-up SLS-T PNC. Reduction of these bonds required extended reducing time, indicating slow kinetics, which is also likely due to the fact that disulfide reduction is reversible. The need for exposure to a reducing environment for extended periods demonstrated in Supplementary Figure 5 may not be met in the DC intracellular trafficking process and may lead to decreased ability to break-up SLS-T PNC.

While PNC essentially serve as the antigen and adjuvant, co-administration of adjuvants is common, and we included the SIINFEKL + poly(I:C) group as a positive control, and also to compare intrinsic vs. extrinsic adjuvant approaches. Poly(I:C) is a synthetic dsRNA analog commonly used as a pathogen-associated molecular pattern (PAMP) adjuvant to non-specifically induce elements of host defense mechanisms associated with viral infection (63). It induces immune signaling associated with cytotoxic T lymphocyte (CTL) responses for intracellular antigens, but also induces innate immune responses similar to many non-specific PAMPs. Poly(I:C) has previously been evaluated with SIINFEKL as a standard method for improving SIINFEKL-specific immune responses since SIINFEKL is an MHC I epitope (21). APCs receive both the antigen and “adjuvant” signal simultaneously from PNC, unlike co-administration of soluble SIINFEKL and poly(I:C), which do not necessarily reach the same cells (64–66). Supplementary Figure 9 corroborates this phenomenon, as significantly more lymphatic DCs that received PNC and upregulated CD86 also presented SIINFEKL and significantly more lymphatic DCs that received PNC and presented SIINFEKL also upregulated CD86. Neither of these combinations was seen for SIINFEKL + Poly(I:C). Furthermore, significantly more lymphatic DCs with CD86 upregulation that received SIINFEKL + Poly(I:C) did not present SIINFEKL, supporting the disconnection between soluble mixtures of SIINFEKL and Poly(I:C).

**Figure 3:**
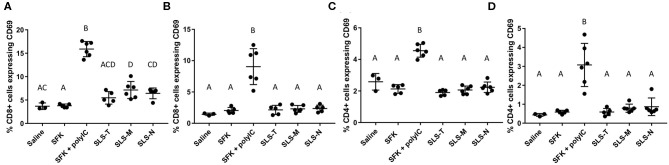
Percent of **(A,B)** CD8+ and **(C,D)** CD4+ T cells in **(A,C)** draining lymph nodes and **(B,D)** spleen expressing early activation marker CD69. *N* = 5–6. Differences between letters show significant differences between samples (*p* < 0.05). Samples that share the same letter are not statistically different from each other.

Contrary to lymph node DCs, splenic DCs only showed significant maturation and MHC I-SIINFEKL presentation when administered soluble SIINFEKL alone (Figure 2B). This could be due to the different trafficking properties of PNC and soluble peptide observed in the biodistribution study discussed below. Antigen in PNC form diffused slower and was trafficked to draining lymph nodes, likely by DCs. Soluble SIINFEKL passively diffused in limited amounts to the spleen, where it may have directly attached to MHC I on DC surfaces without being internalized and presented (67, 68). While MHC I/SIINFEKL and CD86 co-expression is high in the spleen for this soluble group, the low levels of T cell activation in the spleen shown in Figure 3B suggest that DC presentation and signaling were still ineffective at inducing antigen-specific T cell responses. The addition of adjuvant in the SIINFEKL + poly(I:C) group may also affect DC trafficking, resulting in less localization in the spleen. Poly(I:C) is negatively charged, and like other nucleic acid adjuvants, may have the propensity to aggregate (69, 70). Evidence of small aggregates in the soluble SIINFEKL + poly(I:C) were seen after combining adjuvant with antigen. This observation supports that SIINFEKL + poly(I:C) may not represent a completely soluble antigen formulation, resulting in different trafficking properties than those of soluble peptide alone observed in the biodistribution study. Furthermore, adjuvants can cause inflammation which increases lymphatic drainage and affects overall antigen diffusion and transport (71).

MHC II is another DC maturation marker, which presents exogenous antigen for CD4+ T cell activation (30). Lymph node DCs from mice administered SLS-T and SLS-M PNC, though not SLS-N PNC, showed upregulation of MHC II compared to soluble SIINFEKL or and saline (Figure 2C). Upregulation of MHC II in SLS-T and SLS-M groups did not occur at levels that resulted in increased CD4+ activation (Figure 3C). Additionally, upregulation of MHC II is also associated with a reduction in antigen processing, which may have contributed to reduced SIINFEKL presentation in the SLS-T and SLS-M groups (72). No groups displayed significant MHC II expression in the spleen (Supplementary Figure 11). While general DC maturation can be beneficial, MHC II upregulation does not indicate antigen-specific presentation or maturation due to intracellular signaling of an endogenous MHC I antigen such as SIINFEKL. The differences in MHC II upregulation between different PNC types may indicate different signaling in DCs due to different interactions between the PNC and DCs. Different levels of internalization different mechanisms of internalization can affect DC presentation and maturation profiles and overall immune cell response (73). *In vitro* internalization studies demonstrate that all peptide and PNC formulations are internalized by DCs in significant amounts (Supplementary Figure 10). However, the mechanism of internalization may be different for different formulations (39). Several nanoparticle characteristics, including size, shape, surface charge, and hydrophobicity, have been shown to affect mechanisms of internalization, processing, and maturation in DCs (73). SLS-T, SLS-M, and SLS-N PNC are formed via different crosslinking chemistries, and, therefore, differences in surface chemistry and degradability could affect the mechanisms of DC antigen processing and maturation.

#### T Cell Activation

In analyzing the next step in the adaptive immune response process, differences in T cell activation were also observed. CD8+ and CD4+ T cells harvested from lymph nodes and spleens were analyzed for upregulation of CD69, an early activation marker indicatory of T cell proliferation and retention of lymphocytes in antigen-resident tissues (74). Figures 3A,B shows that small, but statistically significant, increases in CD8+/CD69+ T cells were seen only in lymph nodes of mice given SLS-M and SLS-N PNC compared to soluble SIINFEKL. The modest upregulation of CD69 seen only in the lymph nodes for PNC groups correlated with the enhancement of DC presentation of SIINFEKL and maturation in the lymph nodes. In contrast, CD69 expression in CD8+ T cells increased considerably in both the draining lymph nodes and spleen of mice that received SIINFEKL + poly(I:C) adjuvant, despite low maturation levels in SIINFEKL-presenting DCs. Furthermore, CD4+ upregulation of CD69 was observed in lymph nodes and spleen only in the SIINFEKL + poly(I:C) group (Figures 3C,D). These results suggest that non-specific T cell activation occurred in this group due to the uncoupled co-administration of antigen and adjuvant, the nature of poly(I:C) function, or both.

**Figure 4:**
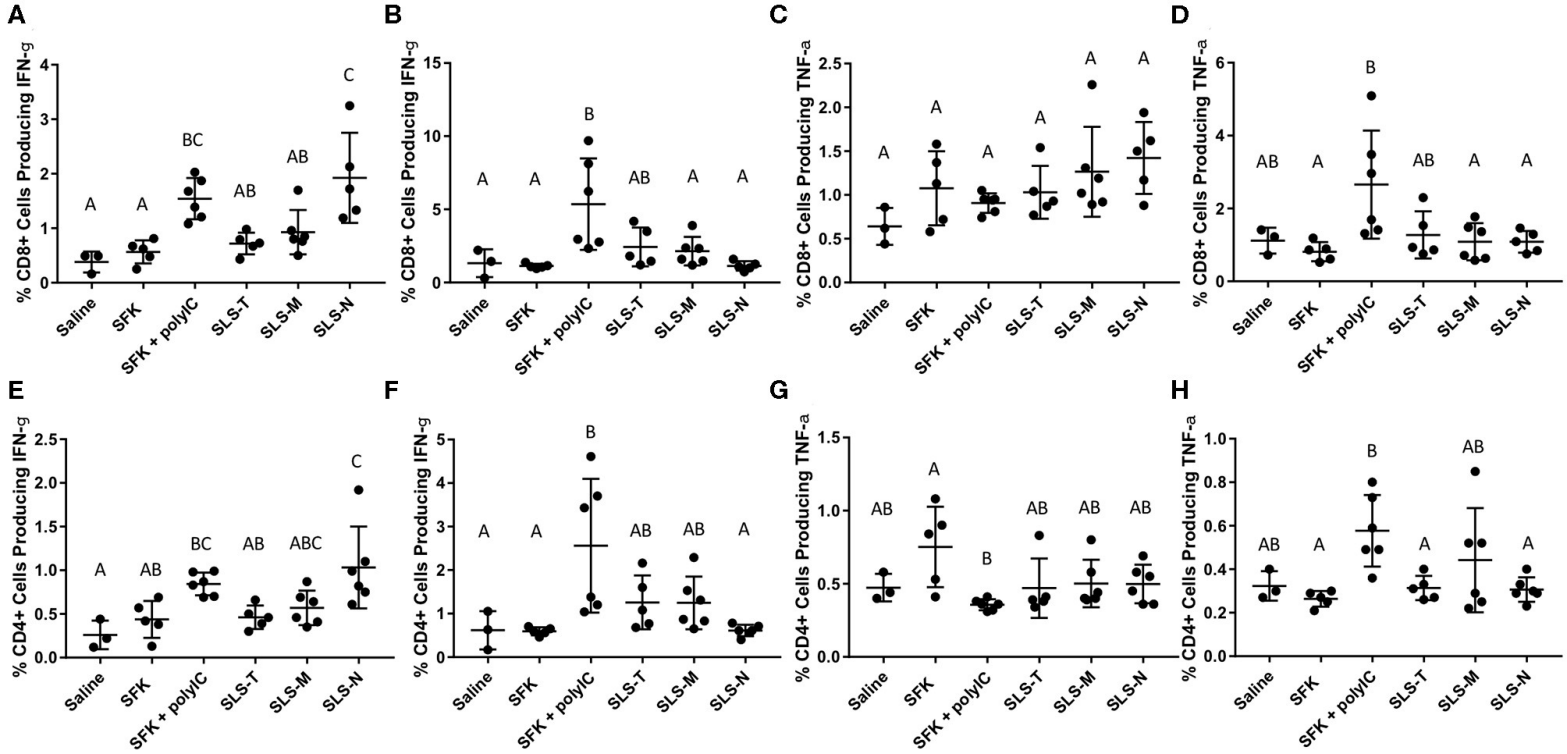
**(A–D)** Percent CD8+ T cells producing **(A,B)** IFN-g and **(C,D)** TNF-a in **(A,C)** axillary/brachial lymph nodes and **(B,D)** spleens when re-stimulated with 1 mg/ml SIINFEKL. **(E–H)** Percent CD4+ T cells producing **(E,F)** IFN-g and **(G,H)** TNF-a in **(E,G)** axillary/brachial lymph nodes and **(F,H)** spleens when re-stimulated with 1 mg/ml SIINFEKL. *N* = 5–6. Differences between letters show significant differences between samples (*p* < 0.05). Samples that share the same letter are not statistically different from each other.

To more directly evaluate SIINFEKL-specific T cell activation, lymphocytes harvested from draining lymph nodes and spleens were re-stimulated *ex vivo* with SIINFEKL peptide and assessed for intracellular cytokines IFN-γ and TNF-α. The intracellular IFN-γ results for CD8+ T cells corroborate the DC and CD69 data that SLS-N PNC have the best conversion of DC presentation into activated, antigen-specific T cells. Figure 4A shows that significantly more CD8+ T cells from mice that received SLS-N PNC produced IFN-γ when re-stimulated with SIINFEKL than those that received soluble SIINFEKL, SLS-T, or SLS-M PNC. Despite the differences between the SLS PNC groups, all three SLS PNC groups without adjuvant displayed statistically similar IFN-γ levels as the SIINFEKL + poly(I:C) group. While the CD69 measurement was not connected to antigen specificity, cytokine production was measured after re-stimulation with SIINFEKL. CD69 upregulation in the SIINFEKL + poly(I:C) group was much greater than PNC groups, but the IFN-γ response was similar to PNC, indicating that cytokine production may have been residual from non-specific activation rather than stimulated by SIINFEKL. Therefore, one conclusion is that despite the lower overall immune cell responses to PNC relative to SIINFEKL + poly(I:C), comparable levels of antigen-specific activation occurred. This may be a more desirable outcome in an effort to maintain balance between a strong enough immune response to induce memory cell formation and pathogen protection and overstimulation that causes allergic reaction or other unwanted side effects commonly associated with adjuvants (75–77). IFN-γ is associated with the increase of MHC I expression and APC recruitment, which ultimately improves the specificity of the adaptive immune response by enabling higher antigen presentation levels and increasing the amount of CD8+ T cell activation and proliferation (30). Consistent with other analyses of spleen-resident DCs and T cells, Figures 4B,F demonstrates that there was no IFN-γ production evident in groups that were administered PNC. A significant amount of splenic T cells from the poly(I:C) adjuvanted group, however, did produce IFN-γ when re-stimulated with SIINFEKL. This correlates with the general activation of T cells in the spleen as shown by CD69 upregulation and may be at least partially due to non-specific adjuvant-related activation. CD4+ T cell production of IFN-γ in all groups and locations displayed the same trends as CD8+ production (Figures 4A,B,E,F). CD4+ T cells cannot have a specific immunological response to the SIINFEKL epitope. CD4+ CD69 upregulation, however, did not align with CD8+ T cell trends; only CD8+ T cells showed upregulation of CD69 in PNC groups. This result suggests that bystander CD4+ activation may have occurred, but only in limited occasions when strong signaling from an antigen-specific response (CD8+ activation/IFN-γ production after re-stimulation with SIINFEKL) was present, such as in the wells *ex vivo*. It has been observed that non-specific, naïve CD4+ T cell activation can occur as a result of strong antigen-specific T cell activation (78, 79). This phenomenon involves the phenotypic changes related to effector T cell function, such as cytokine production, without the necessity for TCR signaling, which could explain the observed IFN-γ production in this study despite the lack of a CD4 epitope (80).

TNF-α production was also measured upon re-stimulation of lymphatic and splenic CD8+ and CD4+ T cells with SIINFEKL (Figures 4C,D,G,H). No groups displayed significant TNF-α production except for splenic CD8+ and CD4+ T cells in the SIINFEKL + poly(I:C) group. While TNF-α production is important for the enhancement of immune responses, it is more often associated with innate immune responses, including increased inflammation (30, 71). Inflammation is valuable in recruiting tissue resident lymphocytes to potentially infected areas. However, systemic production results in dangerous side effects, including shock. TNF-α production in the spleen, therefore, may not necessarily be considered an indicator of strong specific immunity, but rather, evidence of strong, non-specific T cell activation induced by poly(I:C) adjuvant, which may not be beneficial. It is notable that despite comparable levels of lymphatic IFN-γ production in SLS PNC and poly(I:C) groups, TNF-α remained low in lymphatic and splenic tissues evaluated from mice administered PNC.

### Biodistribution

To help explain the differences in immune responses observed between PNC and soluble groups and validate expected nanoparticle transport behavior that may contribute to those differences, peptide localization in the lymph nodes and spleen was assessed. Fluorescent-labeled SLS peptide in soluble and SLS-N PNC form were injected the same way as for the immune response study. It was expected that the modified SIINFEKL peptide, SLS, would have very similar diffusion properties to SIINFEKL because of its similar size. TAMRA-labeled SIINFEKL was highly insoluble in aqueous solutions, and the increased number of hydrophilic residues in SLS increased solubility in aqueous solution when fluorescently-labeled. Therefore, TAMRA-labeled SLS peptide was used as a minimally modified representative soluble peptide that ensured the soluble peptide group did not contain peptide aggregates and eliminated the need for additives to increase solubility of TAMRA-labeled SIINFEKL. Similarly, SLS-N PNC were used as a representative PNC group to evaluate biodistribution because PNC of similar size and morphology were expected to have similar biodistribution. Although several physicochemical characteristics can affect trafficking, such as surface charge, many are often correlated with a potential effect on particle size, which is the most well-studied factor in optimizing lymph node trafficking (81–87). Nanoparticles 20–200 nm are reported to be able to passively diffuse to regional lymph nodes whereas 200–2,000 nm particles require active APC transport to lymph nodes (83). The SLS PNC fall between these two size ranges, so both passive and active trafficking could have occurred. PNC have been shown to have different diffusion rates in tissue than soluble peptide (27). While we previously observed longer injection site retention of PNC, which could increase interactions with tissue resident DCs, it is also important that PNC are trafficked into the lymphatic system where they are more likely to initiate T cell activation.

**Figure 5:**
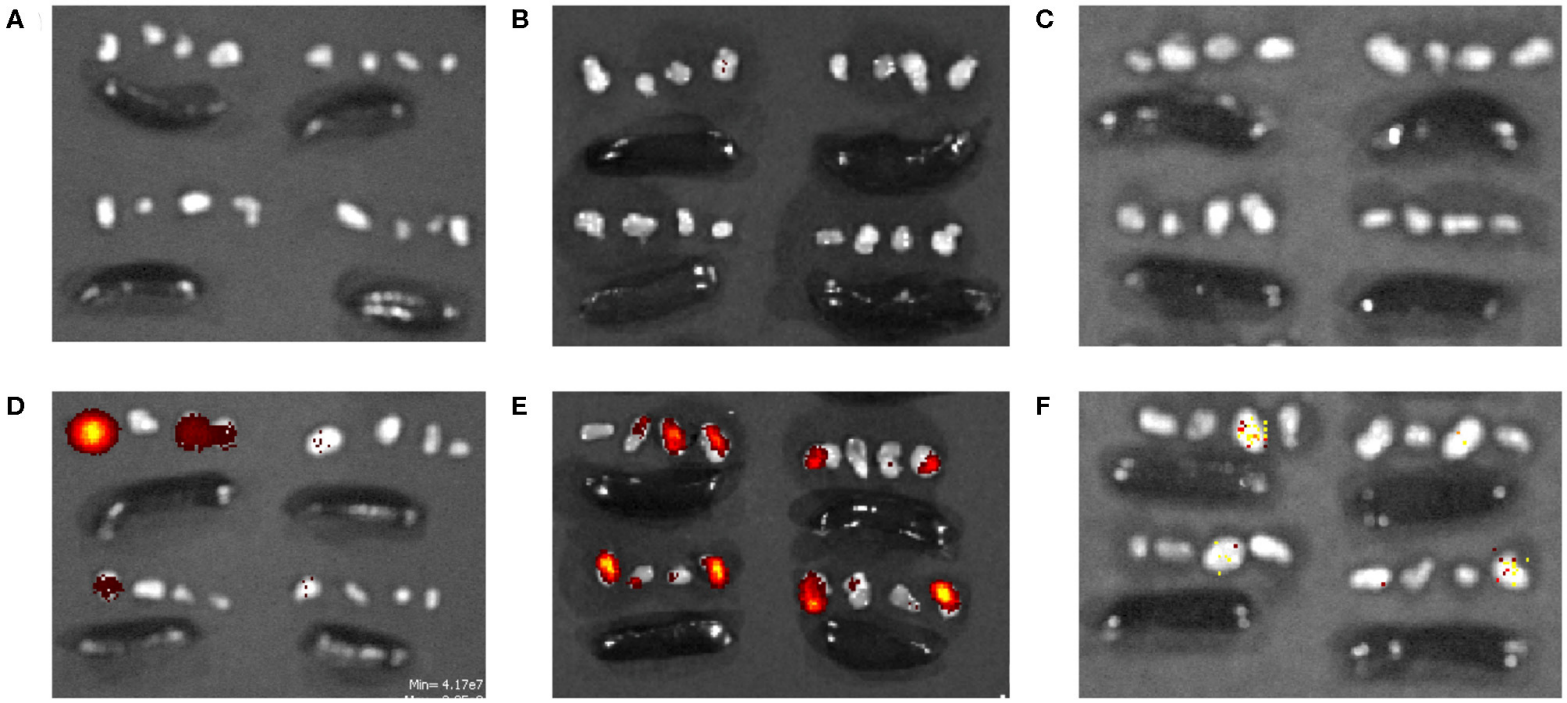
TAMRA-labeled **(A–C)** soluble SLS and **(D–F)** SLS-N PNC in axillary/brachial lymph nodes (groups of 4 small organs) and spleens (large, oblong organs) **(A,D)** 4, **(B,E)** 24, and **(C,F)** 72 h after injection.

**Figure 6:**
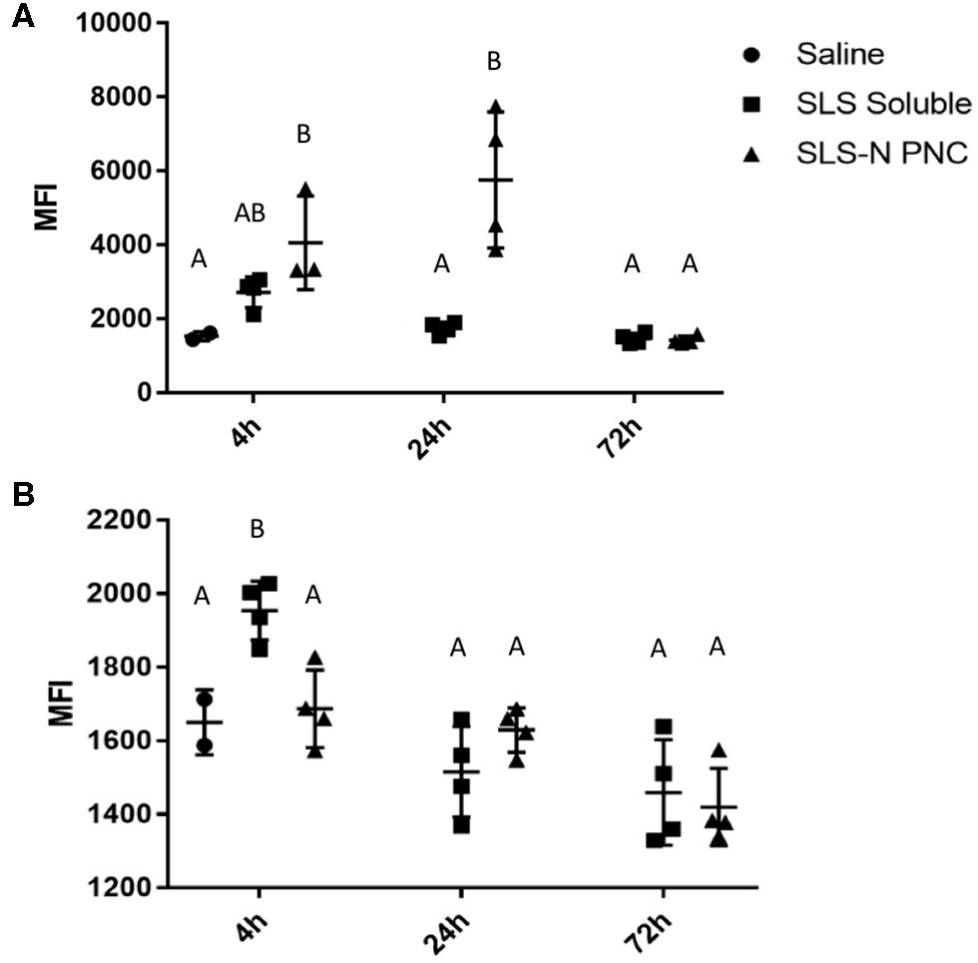
Fluorescent peptide localized in **(A)** draining lymph nodes and **(B)** spleen 4, 24, and 72 h after i.d. injection in both forearms of 10% TAMRA-labeled SLS peptide in soluble or SLS-N PNC form. MFI, Mean Fluorescence Intensity. *N* = 4. Differences between letters show significant differences between samples (*p* < 0.05). Samples that share the same letter are not statistically different from each other.

Peptide localization in axillary and brachial draining lymph nodes and the spleen were evaluated at 4, 24, and 72 h after forearm injections. Figures 5A–C illustrates that soluble fluorescent peptide accumulated in lymph nodes at almost undetectable levels over 72 h. However, as shown in Figures 5D–F, peptide administered in PNC form reached lymph nodes at moderate levels after 4 h and continued to accumulate up to 24 h after injection. After 72 h, low levels of peptide were still detected in some lymph nodes in the SLS-N PNC group. Quantitation of fluorescence in homogenized organs confirmed that a significant amount of peptide was retained in lymph nodes at both 4 and 24 h after injection only in mice that received SLS-N PNC (Figure 6). These results validate several immune response observations. The increased amount of PNC peptide trafficking to draining lymph nodes combined with retention of PNC peptide in the nodes for at least 20 h align with improved DC presentation and maturation as well as T cell activation in these tissues. This correlation between improved immune response due to these trafficking and retention properties is corroborated by several other studies that evaluate biomaterials for increased immunogenicity of subunit vaccines (16, 88, 89).

Although undetectable in fluorescent images, quantitation of peptide in homogenized spleens revealed that significant splenic accumulation of soluble peptide occurred 4 h after injection, but no soluble peptide was detected in the spleen at 24 or 72 h (Figure 6B). These results illustrate that although there is limited passive diffusion or trafficking of soluble antigen through the lymphatic system, this process occurs more quickly and transiently than for PNC. The insufficient residence time of soluble peptide aligned with the similarly limited immune responses observed with soluble peptide administration *in vivo*. There was some evidence of DC presentation and maturation in the spleen, in agreement with low levels of peptide present there, but this ultimately did not result in CD8+ T cell activation. Similarly, soluble peptide that was administered with adjuvant also displayed evidence of immune cell activation in splenic cells. The ability to quantify soluble peptide in the spleen, although transiently, implies that the limited presence of peptide in the lymphatic system can still induce a response when there is significant activation signaling provided by an adjuvant, which was observed in our immune response studies. This observation supports the many instances of increased immune responses with peptide/adjuvant co-administration seen in vaccine formulations (24, 63, 90) It should be noted, however, that while some aspects of adjuvant driven immune responses are beneficial, there are several drawbacks for vaccine safety and control over the immune response (91, 92). These challenges highlight the value of development of biomaterials, such as PNC, which enhance only certain aspects of immune cell activation more associated with antigen-specific adaptive immunity and may be more desirable from a safety standpoint.

## Conclusion

While modifying peptide antigens for biomaterial incorporation has the potential to reduce specificity and the ability of the antigen to interact with immune cells effectively, observations in this work suggest that limited, strategic modifications when combined with biomaterial formation offer benefits that afford similar or better levels of activation against the target antigen. Importantly, the method of incorporation into biomaterials in PNC affected the ability of the material to induce improved immunogenicity at several stages of activation. DC presentation and maturation were significantly improved over soluble antigen for PNC stabilized by amide bonds. This improved antigen-specific response carried through to T cell activation, where antigen-specific CD8+ responses were observed in all SLS PNC at comparable levels to antigen with adjuvant. These results suggest that utilizing design tools to alter antigens in a way that allows several routes of biomaterial incorporation may be highly beneficial so that a method that maximizes the desired response can be identified. More specifically, SLS-N PNC demonstrate the importance of understanding the likely mechanism of material processing and ultimate intracellular fate. Utilizing this amide bond crosslinking formulation, which mimicked foreign antigens in that it was proteolytically cleavable but stable in most other environments, proved to be highly beneficial in achieving the desired increased immune cell response. Additionally, the ability to improve specific responses to a target antigen with comparable levels of activation to an adjuvanted antigen formulation, while maintaining low levels of non-specific activation markers, demonstrates a unique level of control provided by PNC.

More work is needed to further characterize the various modifications and conjugation mechanisms that may be used for different methods of biomaterial incorporation of antigens. However, the knowledge developed in this study provides insight into the immune cell activation processes triggered by biomaterials depending on their formulation. These results offer a guide both for future PNC synthesis and antigen incorporation methods into a variety engineered peptide biomaterial subunit vaccines, as they demonstrate potential approaches to systematically design biomaterials to control and maximize the specific immune cell responses desired.

## Data Availability Statement

The raw data supporting the conclusions of this article will be made available by the authors, without undue reservation.

## Ethics Statement

The animal studies were reviewed and approved by Georgia Institute of Technology Institutional Animal Care and Use Committee.

## Author Contributions

AT and JC contributed conception and design of the study. AT conducted experiments, organized results, and performed statistical analyses. KW and AP contributed design of yield characterization. KW performed experiments and analysis and contributed manuscript text for NMR yield characterization. AT drafted the manuscript. JC and AP edited the manuscript. All authors contributed to manuscript revision, read, and approved the submitted version.

## Conflict of Interest

The authors declare that the research was conducted in the absence of any commercial or financial relationships that could be construed as a potential conflict of interest.
