# Supplementary material for: Rational Design of Antigen Incorporation Into Subunit Vaccine Biomaterials Can Enhance Antigen-Specific Immune Responses
Source: Front Immunol. 2020 Jul 21;11:1547. doi: 10.3389/fimmu.2020.01547 (PMC7396695; doi:10.3389/fimmu.2020.01547)
Supplement: Supplementary file 1 [file Data_Sheet_1.pdf]

frontiers

Supplementary Material

Supplementary Table S1. Desolvation conditions and crosslinker amounts for each PNC type.

PNC	Crosslinker	Crosslinker Reactive Group (Molar Excess)	Crosslinker Amount (mg)	Crosslinker Volume (µl)	DEE Volume (µl)	Reaction Time (min)
SLS-T	Tri-thiol	50	3.22	2.66	500	50
SLS-M	Tri-maleimide	5	0.210	21.0	800	60
SLS-N	Tri-NHS	0.5	0.052	5.20	500	60

Supplementary Table S2. Zetasizer Nano ZS dynamic light scattering measurement settings.
*Based on starting peptide concentration

Concentration (mg/ml)*	Resuspension Medium	Volume (µL)	Measurements Per Sample Run	Laser Wavelength (nm)	Scattering Angle (˚)
1	MQ Water	70	3	633	173

Cuvette Type	Measurement Temperature (˚C)	Refractive Index (Particle)	Refractive Index (Medium)	Viscosity (cP)
ZEN 0040	25	1.45	1.33	0.8872

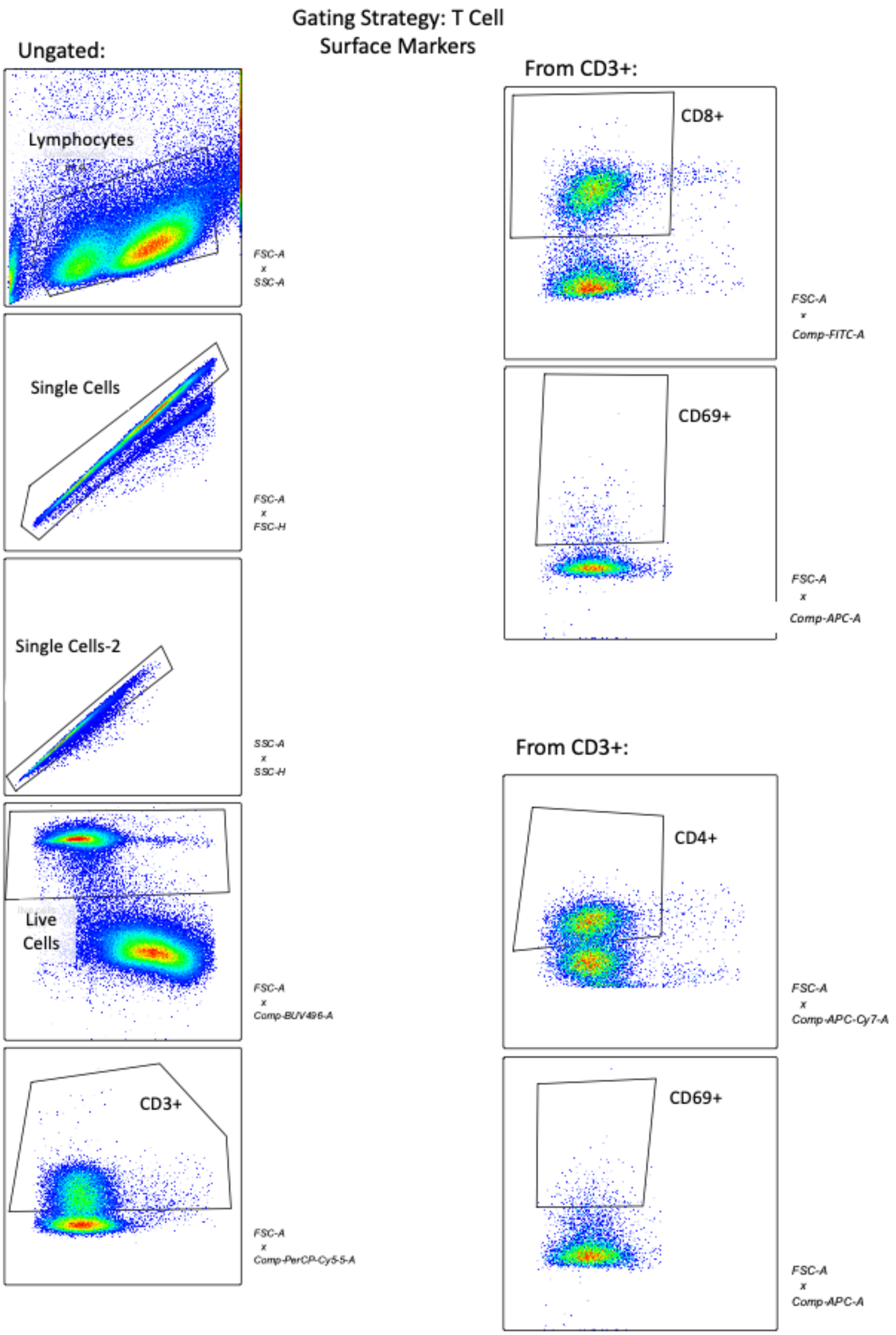

Supplementary Figure S1. Gating strategy used for quantifying T cells with CD69 surface marker expression.

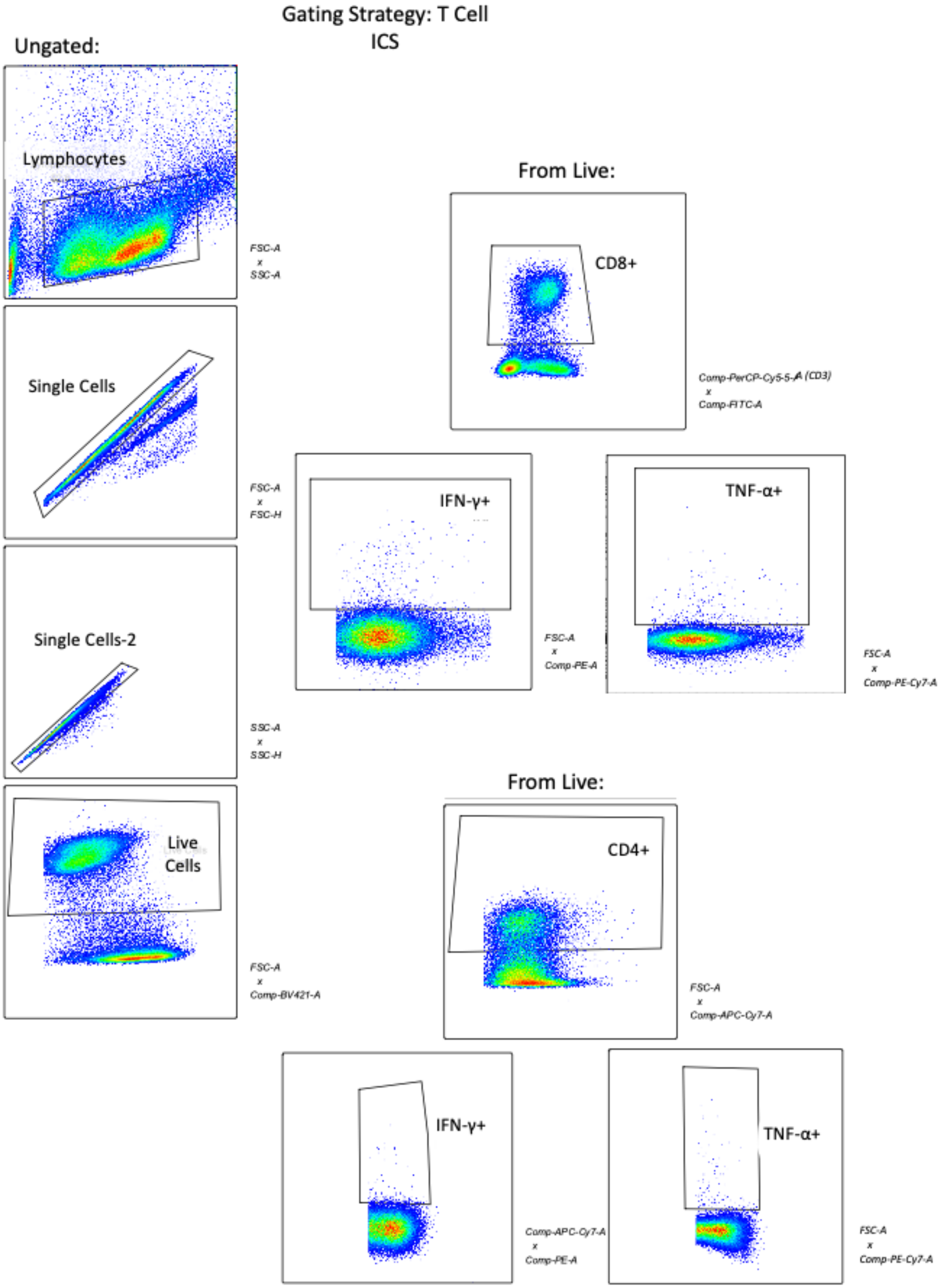

Supplementary Figure S2. Gating strategy used for quantifying T cells with IFN-γ and TNF-α cytokine production.

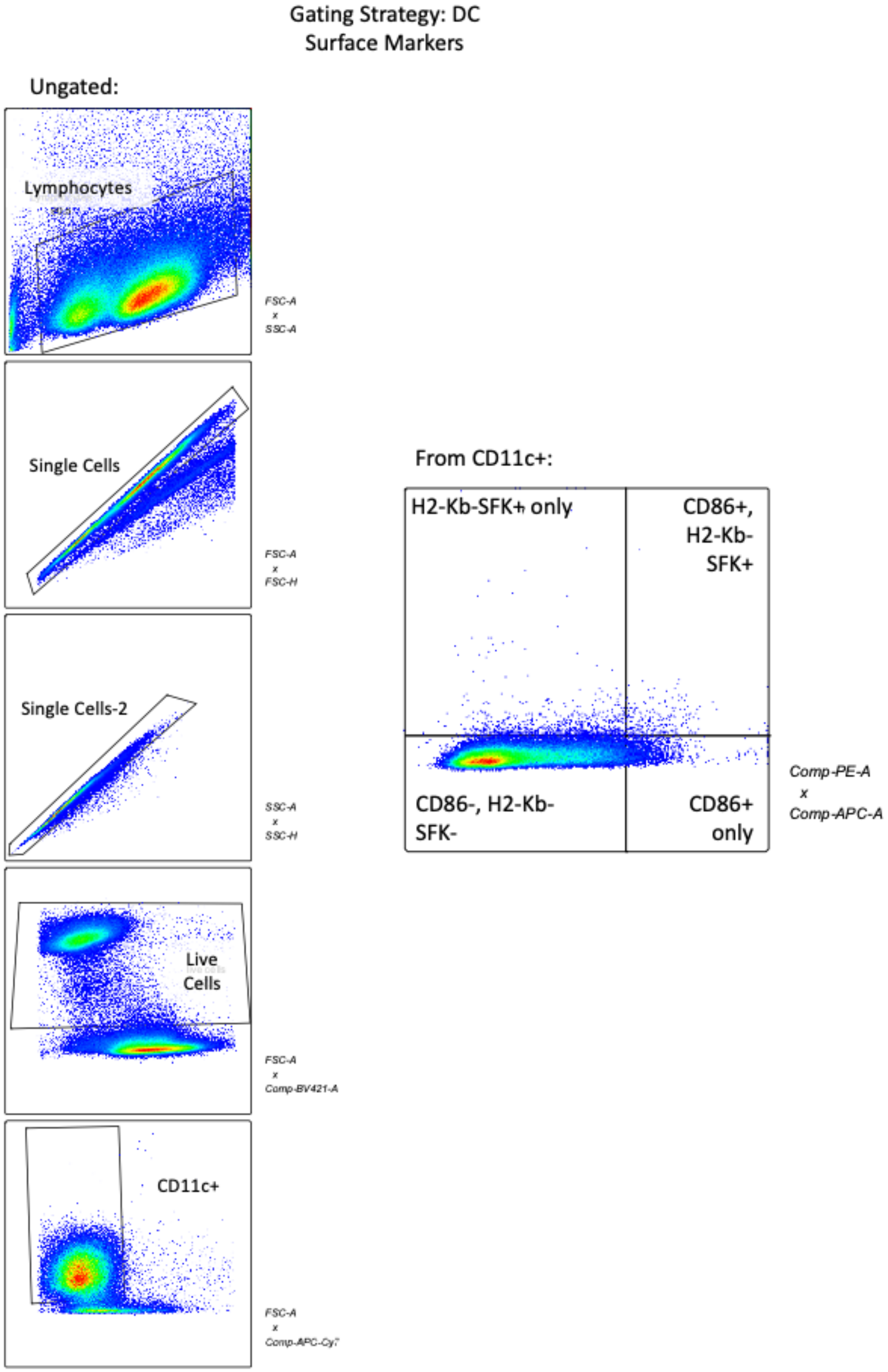

Supplementary Figure S3. Gating strategy used for quantifying DCs with CD86 surface marker expression and SIINFEKL presentation on the MHC I haplotype H2-K^{b}.

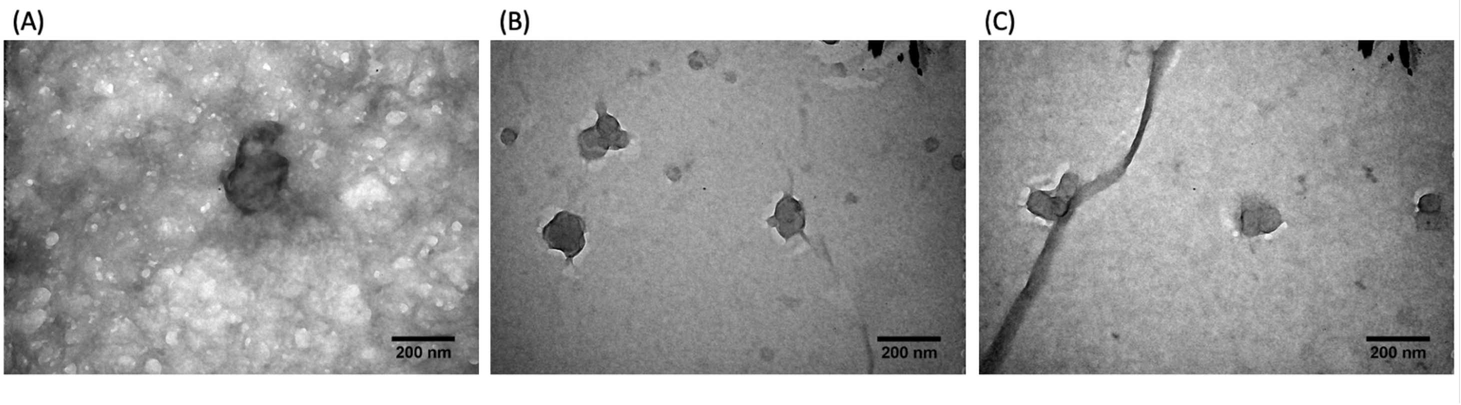

Supplementary Figure S4. TEM images of (A) SLS-T, (B) SLS-M, and (C) SLS-N PNC. Scale bars = 200 nm. Particles were dried on a TEM grid (Carbon Film 300 Mesh, Copper; Electron Microscopy Sciences) overnight. Dried PNC were stained with 1% phosphotungstic acid (PTA) for 15 seconds, washed twice with water, and dried again overnight before imaging using a JEOL 100 CX-II TEM.

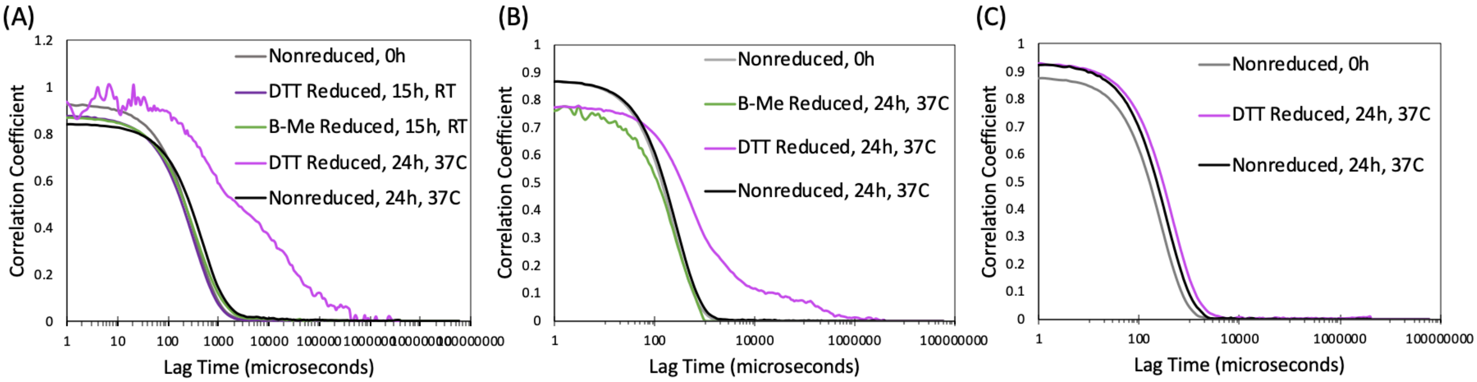

Supplementary Figure S5. (A) SLS-T, (B) SLS-M, and (C) SLS-N PNC breakup via reduction with 5% v/v $\boldsymbol{\beta}$-mercaptoethanol (B-mE) or 100 mM dithiothreitol (DTT) at room temperature (RT) or 37 ˚C. Dynamic light scattering correlogram indicates intact particles for all measurements except when SLS-T or SLS-M particles are reduced with 100 mM DTT for 24 h at 37 ˚C, where correlogram displays poor quality particles with aggregation.

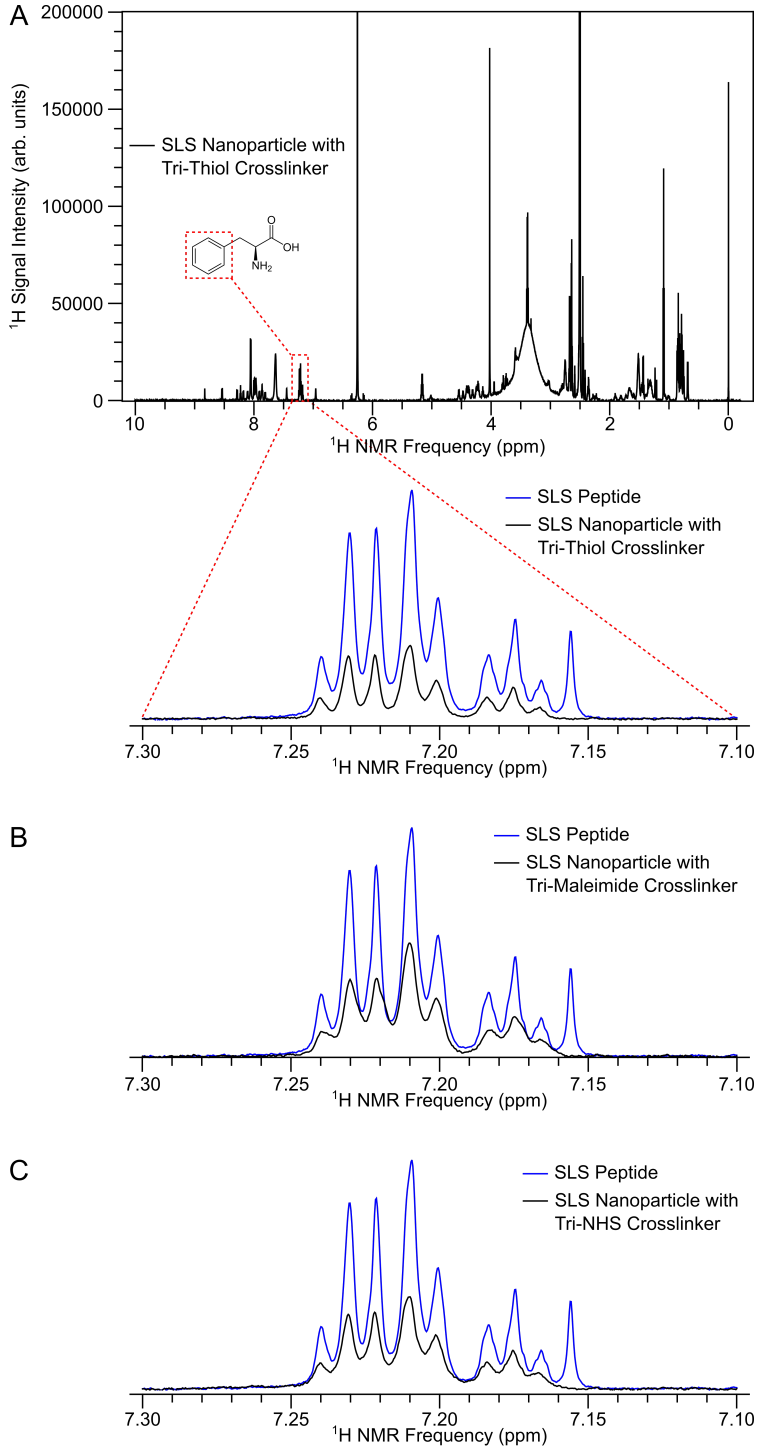

Supplementary Figure S6. ^{1}H NMR spectra of SLS PNC resuspended in deuterated DMSO. (A) Full ^{1}H NMR spectrum of SLS-T PNC with the aromatic protons of the phenylalanine sidechain highlighted in red. Below is a zoomed in overlay of the unassembled SLS peptide (blue) and SLS-T PNC (black) spectra focused on the phenylalanine sidechain region. (B,C) Overlay of the unassembled SLS peptide and (B) SLS-M and (C) SLS-N PNC spectra focused on the phenylalanine sidechain region.

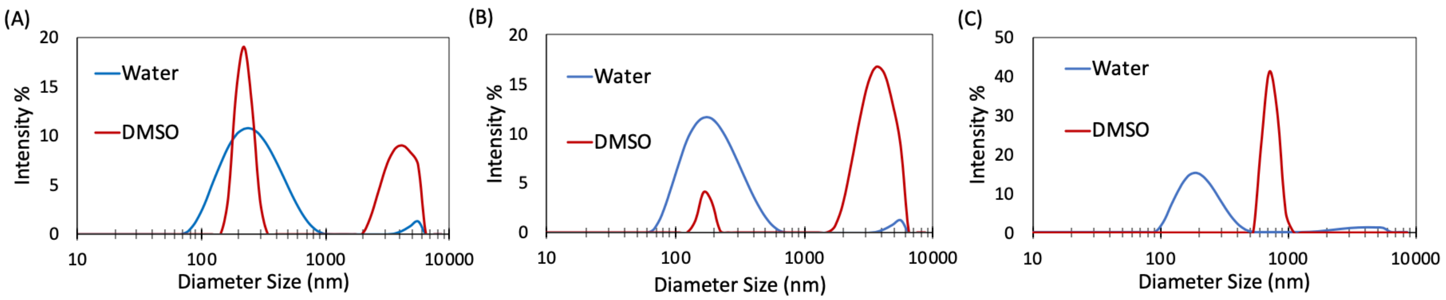

Supplementary Figure S7. Size distribution of (A) SLS-T, (B) SLS-M, and (C) SLS-N PNC measured by dynamic light scattering when suspended in water or DMSO. PNC increase in size when suspended in DMSO. Size increase indicates particle swelling, ensuring sufficient molecular mobility during ^{1}H NMR analysis.

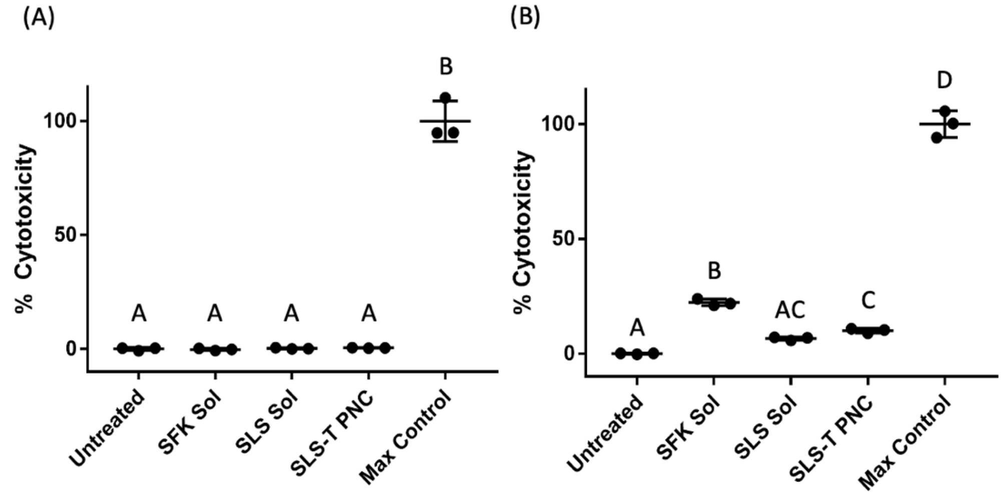

Supplementary Figure S8. Cytotoxicity of SIINFEKL, SLS, and SLS-T PNC in DC2.4 dendritic cell and B3Z T cell hybridomas. No toxicity was observed in DC cell line. Low levels of toxicity were observed with SLS-T PNC. However, higher toxicity was observed in soluble SIINFEKL than the SLS and SLS-T groups, despite being a very common antigen that is widely used *in vivo.* 100 µl/well DCs (100,000 cell/ml) or T cells (100,000 cells/ml) were incubated with 25 µg/ml peptide in soluble or PNC form in supplemented culture medium (RPMI 1640 + L-glutamine + 25 mM HEPES supplemented with 10% heat inactivated fetal bovine serum, 1% penicillin/streptomycin, 50 µM β-mercaptoethanol). After 24 h incubation, cytotoxicity was measured with an LDH Cytotoxicity Assay kit according to manufacturer protocol (Pierce; ThermoFisher). Maximum (Max) control was DC or T cell LDH production after 30 min incubation with 1X lysis buffer. 100% toxicity was determined from Max Control. N=3. Differences between letters show significant differences between samples ($p < 0.01$). Samples that share the same letter are not statistically different from each other.

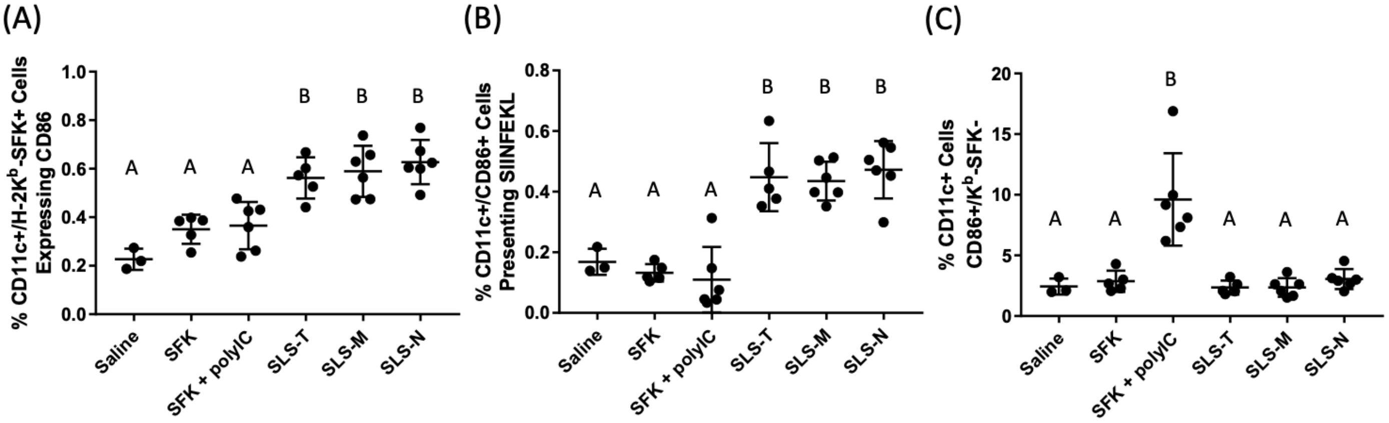

Supplementary Figure S9. (A) Percent of CD11c+/H-2K^{b}-SFK+ DCs in axillary/brachial lymph nodes that also express CD86 maturation marker. (B) Percent of CD11c+/CD86 + maturing DCs in axillary/brachial lymph nodes that also present SIINFEKL. (C) Percent of CD11c+ DCs in axillary/brachial lymph nodes that are CD86+/H-2K^{b}-SFK-. Of the DCs presenting SIINFEKL, only PNC induce significant maturation (CD86 expression). Similarly, of the CD86+ maturing DCs, only PNC groups showed a significant percentage of SIINFEKL presentation. Nonspecific maturation (CD86 expression) without SIINFEKL presentation occurs at high levels only in the poly(I:C) adjuvanted SIINFEKL group. N=5-6. Differences between letters show significant differences between samples ($p < 0.05$). Samples that share the same letter are not statistically different from each other.

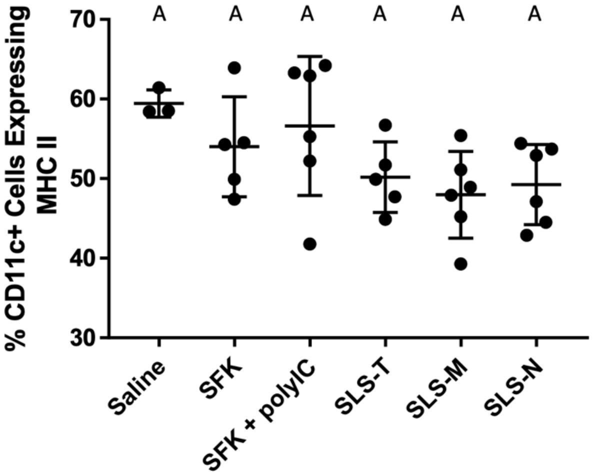

Supplementary Figure S10. Percent of CD11c+ cells in spleen expressing MHC II. N=5-6. Differences between letters show significant differences between samples ($p < 0.05$). Samples that share the same letter are not statistically different from each other.

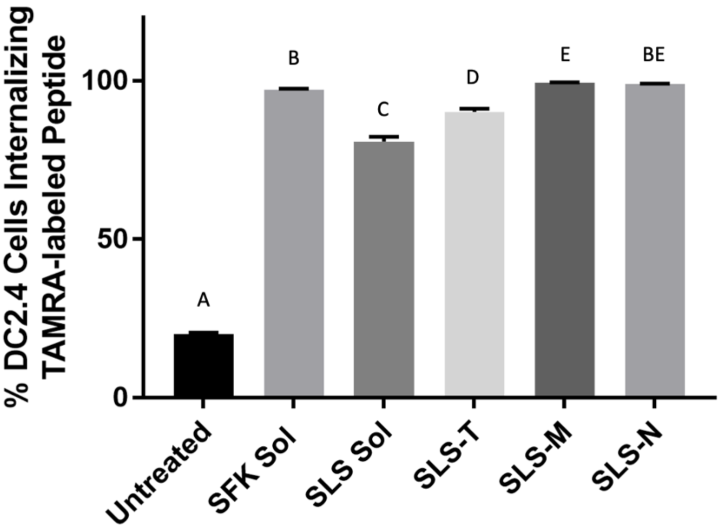

Supplementary Figure S11. DC2.4 dendritic cell line internalization of 10% TAMRA-labeled peptide in soluble and PNC form. All treatment groups displayed significant uptake. Increased uptake of soluble SIINFEKL peptide could be due to aggregation of TAMRA-labelled SIINFEKL in aqueous media, which did not occur for TAMRA-labelled SLS. Slight differences in PNC uptake may reflect differences in PNCs detectable by cells, or slight variations in the efficiency of TAMRA-labelled SLS incorporation in PNCs. 50,000 DC2.4 cells/well were seeded in a 96 well V-bottom plate in culture medium (RPMI 1640 + L-glutamine + 25 mM HEPES supplemented with 10% heat inactivated fetal bovine serum, 1% penicillin/streptomycin, 50 μM β-mercaptoethanol). For 24 h at 37 °C and 5% CO_2, DCs were incubated with 25 μg/ml of the indicated antigen formulation with 10% of the peptide TAMRA-labelled. Cells were centrifuged in the plate at 300 g for 5 mins, washed once with 100 μl acid wash (0.2 M glycine, 0.15 M NaCl, pH 3.1), washed twice with 100 μl PBS, and resuspended in 200 μl 1% BSA in PBS for analysis. Flow cytometry was performed with Beckman Coulter CytoFLEX and collected 5000 cells/well. N=3. Differences between letters show significant differences between samples ($p < 0.05$). Samples that share the same letter are not statistically different from each other.
